# Lymphopenia as a Predictor for Adverse Clinical Outcomes in Hospitalized Patients with COVID-19: A Single Center Retrospective Study of 4485 Cases

**DOI:** 10.3390/jcm11030700

**Published:** 2022-01-28

**Authors:** Jianli Niu, Candice Sareli, Daniel Mayer, Alvaro Visbal, Aharon Sareli

**Affiliations:** 1 Office of Human Research, Memorial Healthcare System, Hollywood, FL 33021, USA; JNiu@mhs.net (J.N.); CSareli@mhs.net (C.S.); 2 Adult Critical Care Service, Memorial Healthcare System, Hollywood, FL 33021, USA; DMayer@mhs.net (D.M.); AVisbal@mhs.net (A.V.)

**Keywords:** lymphocyte count, lymphopenia, adverse clinical outcomes, COVID-19, restricted cubic splines, receiver operating characteristic curves

## Abstract

Lymphopenia is commonly present in patients with COVID-19. We sought to determine if lymphopenia on admission predicts COVID-19 clinical outcomes. A retrospective chart review was performed on 4485 patients with laboratory-confirmed COVID-19, who were admitted to the hospital. Of those, 2409 (57.3%) patients presented with lymphopenia (absolute lymphocyte count < 1.1 × 10^9^/L) on admission, and had higher incidences of ICU admission (17.9% versus 9.5%, *p* < 0.001), invasive mechanical ventilation (14.4% versus 6.5%, *p* < 0.001), dialysis (3.4% versus 1.8%, *p* < 0.001) and in-hospital mortality (16.6% versus 6.6%, *p* < 0.001), with multivariable-adjusted odds ratios of 1.86 (95% confidence interval [CI], 1.55–2.25), 2.09 (95% CI, 1.69–2.59), 1.77 (95% CI, 1.19–2.68), and 2.19 (95% CI 1.76–2.72) for the corresponding outcomes, respectively, compared to those without lymphopenia. The restricted cubic spline models showed a non-linear relationship between lymphocyte count and adverse outcomes, with an increase in the risk of adverse outcomes for lower lymphocyte counts in patients with lymphopenia. The predictive powers of lymphopenia, expressed as areas under the receiver operating characteristic curves, were 0.68, 0.69, 0.78, and 0.79 for the corresponding adverse outcomes, respectively, after incorporating age, gender, race, and comorbidities. In conclusion, lymphopenia is a useful metric in prognosticating outcomes in hospitalized COVID-19 patients.

## 1. Introduction

The COVID-19 pandemic, caused by the severe acute respiratory syndrome coronavirus 2 (SARS-CoV-2), remains a global public healthcare challenge. The clinical course of COVID-19 ranges from asymptomatic disease to critical illness and death [1,2,3,4]. The risk of poor outcomes associated with COVID-19 appears to increase with old age, male gender, smoking status and the coexistence of comorbidities such as obesity, hypertension, diabetes, cardiovascular disease, chronic kidney disease, chronic obstructive pulmonary disease, and malignancy [3,4,5,6,7]. Several inflammatory response markers including procalcitonin, serum ferritin, C-reactive protein (CRP), D-dimer, and interleukin-6 (IL-6), which have been found to correlate significantly with severity of disease and poorer outcomes of COVID-19 patients [8,9]. However, the use of common clinical characteristics and serum biomarkers has significant limitations in reliably predicting clinical outcomes such as admission intensive care unit (ICU), need for mechanical ventilation, and death in hospitalized COVID-19 patients [10,11]. Given the recurrence of high prevalence SARS-CoV-2 waves in the United States, prognostication and risk assessments of adverse outcomes in COVID-19 patients are key priorities.

Lymphocytes play a pivotal role in the immune response against viral infections [12]. A decrease in lymphocyte counts has been described in many respiratory infections caused by viruses [13,14]. Importantly, a low lymphocyte count has been proposed as a worse prognostic marker in patients with influenza virus infection [15,16], chronic obstructive pulmonary disease [17], heart diseases [18,19], and malignancy [20]. In patients infected with SARS-CoV-2, a low lymphocyte count in peripheral blood is a common clinical feature, particularly in patients with severe COVID-19 [2,21,22]. Thus far, several studies have suggested that the degree of lymphocyte count reduction correlates with illness severity in patients with COVID-19 [8,22,23]. It remains uncertain whether a low lymphocyte count at admission in COVID-19 patients may signify a risk of subsequent adverse clinical consequences. Furthermore, the extent to which lymphopenia is associated with poor clinical outcomes merits further definition. Herein we performed a retrospective observational study of a large cohort of adult COVID-19 patients hospitalized at the Memorial Healthcare System, Hollywood, Florida, between 7 March 2020 and 18 January 2021, to test the hypothesis that lymphopenia has the prognostic capacity for adverse clinical outcomes in hospitalized COVID-19 patients. We investigated the relationship between the continuous changes of absolute lymphocyte count (ALC) on admission and the risk of developing various adverse clinical outcomes such as requirement for admission to the ICU, use of invasive mechanical ventilation, requirement of dialysis due to acute kidney injury (AKI), and in-hospital mortality in COVID-19 patients. We also explored the correlations between the lymphopenia and serum inflammatory biomarkers in hospitalized COVID-19 patients. In addition, we tested whether the prognostic capacity of lymphopenia is strengthened by the incorporation of other known risk factors associated with poor outcomes in COVID-19 patients. 

## 2. Materials and Methods

### 2.1. Study Population and Design

This was a single center, retrospective, observational study of consecutive patients who were hospitalized with a diagnosis of COVID-19 at any of the four adult hospitals of the Memorial Healthcare System (MHS) in Hollywood, Florida from 7 March 2020 to 18 January 2021. COVID-19 diagnosis in laboratory-confirmed patients were based on polymerase chain reaction (PCR) tests. Patients with positive SARS-CoV-2 PCR tests were included in the study. Patients were excluded from the study if they were (1) age under 18 years; (2) missing absolute lymphocyte count; (3) hospitalization was less than 24 h; (4) known HIV and/or hematologic disease; and (4) ALC > 5.0 × 10^9^/L. This study was approved by the Institutional Review Board of MHS (protocol No. MHS.2020.065). Written informed consent was waived as this was a retrospective analysis of existing data.

### 2.2. Data Collection

Data were obtained from patients’ electronic medical records. Demographic characteristics included age, gender, race/ethnicity, and underlying conditions such as obesity (defined as a calculated body mass index ≥ 30 kg/m^2^), smoking status, hypertension, diabetes, chronic obstructive pulmonary disease (COPD), coronary heart disease, chronic kidney disease, and malignancy. Laboratory results, including ALC and serum levels for CRP, D-dimers, IL-6, lactate dehydrogenase (LDH), and creatinine obtained within 24 h of hospitalization were used in the study.

All patients in the study were followed from admission to death or to discharge from the hospital. For patients with multiple admissions during the study period, the most severe outcome was assigned. The primary outcome of this study was all-cause mortality during hospitalization. Secondary outcomes included requirement for admission to the ICU, use of invasive mechanical ventilation, and requirement of dialysis due to AKI. Lymphopenia was defined as an ALC < 1.1 × 10^9^/L (1100 cells/mm^3^) based on a previous meta-analysis showing a consistent outcome in four studies using the same cutoff point [21].

### 2.3. Statistical Analysis

Descriptive statistics were used to summarize characteristics and outcomes from the study population. Continuous variables were presented using median and interquartile range (IQR) or mean ± standard deviation (SD), whereas categorical variables were presented using absolute number and frequencies (%). Comparisons between groups were performed using independent sample *t*-tests, Mann–Whitney U-tests, Chi-square or Fischer’s exact tests, as appropriate. The Spearman rank test was used to analyze the correlations between ALC and serum levels of CRP, IL-6, LDH, D-dimer, and creatinine. The Kendall’s tau-b test was performed to measure the correlations between ALC and the adverse outcomes of interest, including in-hospital mortality, requirement for ICU admission, use of invasive mechanical ventilation, and requirement for dialysis.

Multivariable logistic regression models were constructed to estimate the odds ratios (ORs), adjusted ORs and their 95% confidence intervals (CIs) for association of lymphopenia with the four outcomes of interest. Covariates for the various models were chosen based on a prior review of the literature and clinical judgment, focusing on variables that might be expected to confound the lymphopenia-risk relationship. Models were initially adjusted for age and gender (model A), and subsequently additionally adjusted for the known risk factors including race/ethnicity, hypertension, diabetes, COPD, chronic kidney disease, coronary artery disease, malignancy, obesity, and smoking (model B). Restricted cubic splines (RCS) [24] with four knots at the 25th, 50th, 75th, and 95th percentiles of ALC distribution were incorporated into the logistic models to flexibly estimate risks for the outcomes of interest based on the continuous changes of lymphocyte counts. The results were visually presented with graphs by entering the lymphocyte count as a continuous variable into the logistic regression analysis, with a reference point at 1.1 × 10^9^/L. These analyses were adjusted for the variables in model B. The receiver operating characteristic (ROC) curves were then determined to determine the prognostic power of lymphopenia alone or adjusted with other clinical variables for predicting the outcomes of interest. Differences in ROC area under curve (AUC) were tested using the method of Delong et al. [25]. Statistical analyses were performed using the Statistical Package for the Social Sciences (SPSS version 27; Chicago, IL, USA), STATA version 15 (StataCorp, LLC, College Station, TX, USA). A *p* value of less than 0.05 was considered statistically significant.

## 3. Results

**Figure 1:**
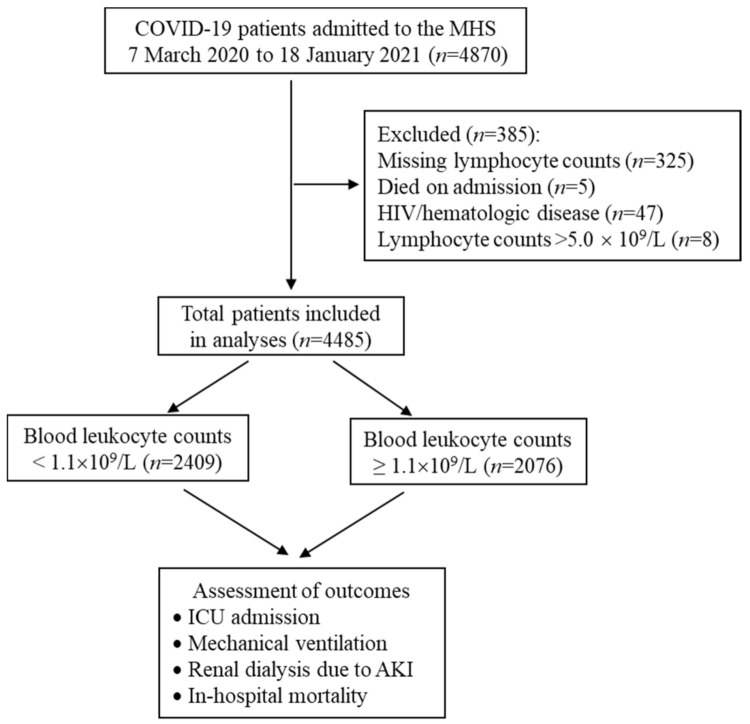
Flow chart of study population and clinical course assessment. Lymphocytes were determined using enrollment complete blood counts. ICU, intensive care unit; AKI, acute kidney failure.

Between 7 March 2020 and 18 January 2021, a total of 4870 adult patients were admitted to the MHS for COVID-19. After excluding five patients who died during the first 24 h after admission, 47 patients who had HIV, 325 patients with unavailable lymphocyte counts in the electronic medical record, and eight patients with lymphocyte counts > 5.0 × 10^9^/L (considering that they could suffer from a concomitant lymphoproliferative disorder), we included 4485 patients with COVID-19 in the final analysis (Figure 1).

### 3.1. Patient Characteristics Associated with Lymphopenia

At the time of hospital admission, the mean absolute lymphocyte count of the study population was 1.18 (SD, 0.62) × 10^9^/L with a median of 1.1 (IQR, 0.7–1.5) × 10^9^/L. Table 1 summarizes the baseline characteristics of 4485 patients stratified by lymphopenia (defined as an ALC < 1.1 × 10^9^/L). A total of 2409 (53.7%) patients had lymphopenia, and 2076 (46.3%) patients had no lymphopenia. Compared with patients without lymphopenia, patients with lymphopenia tended to be significantly older, more male and Hispanic. This subset of patients also had a significantly higher comorbidity profile including hypertension, chronic obstructive pulmonary disease, chronic kidney disease, coronary artery disease, obesity, and smoking. Diabetes mellitus and malignancy were two comorbidities that did not significantly differ between patients with lymphopenia and without. Laboratory testing on admission revealed that patients with lymphopenia had higher levels of CRP, IL-6, LDH, and creatinine compared to patients without lymphopenia. 

### 3.2. Relationships between Lymphocyte Counts and Adverse Clinical Outcomes 

**Figure 2:**
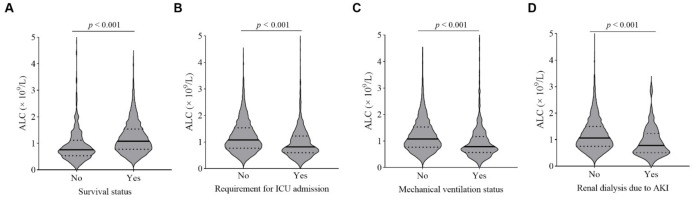
Relationships of ALCs on hospital admission with adverse clinical outcomes in patients with COVID-19. Violin plots of absolute lymphocyte counts (ALCs) showing significantly lower lymphocyte counts in patients who died (**A**) and those who required ICU admission (**B**), invasive mechanical ventilation (**C**), and dialysis (**D**) than those who were not (*p* < 0.001, Mann–Whitney U-test). Solid black lines indicate medians, and dashed black lines represent quartile ranges.

Figure 2 depicts absolute lymphocyte counts measured on admission and the relationship with adverse clinical outcomes in COVID-19 patients. The lymphocyte counts on admission were significantly lower in patients who died and those who required ICU admission, invasive mechanical ventilation, or dialysis (all *p* < 0.001). A Kendall rank correlation analysis demonstrated an inverse correlation between lymphocyte counts and in-hospital mortality (correlation coefficient, −0.161, *p* < 0.001), ICU admission (correlation coefficient, −0.134, *p* < 0.001), invasive mechanical ventilation (correlation coefficient, −0.133, *p* < 0.001), and need for dialysis (correlation coefficient, −0.068, *p* < 0.001). 

Elevated serum levels of CRP, D-dimer, IL-6, LDH, and creatinine have been associated with disease progression and poor clinical outcomes in COVID-19 patients [9,11]. We analyzed the relationship between lymphocyte counts and the serum levels of CRP, D-dimer, IL-6, LDH, and creatinine. Utilizing Spearman correlation analysis, there was a statistically significant inverse relationship between lymphocyte counts and the serum levels of CRP (correlation coefficient, −0.130, *p* < 0.001), D-dimer (correlation coefficient, −0.041, *p* = 0.014), IL-6 (correlation coefficient, −0.115, *p* = 0.001), LDH (correlation coefficient, −0.163, *p* < 0.001), and creatinine (correlation coefficient, −0.092, *p* < 0.001), respectively, suggesting a clear relationship of a lower lymphocyte count at the time of hospital admission with high levels of CRP, D-dimer, IL-6, LDH, and creatinine in COVID-19 patients.

### 3.3. Lymphopenia Is Associated with Higher Incidences of Adverse Clinical Outcomes

**Figure 3:**
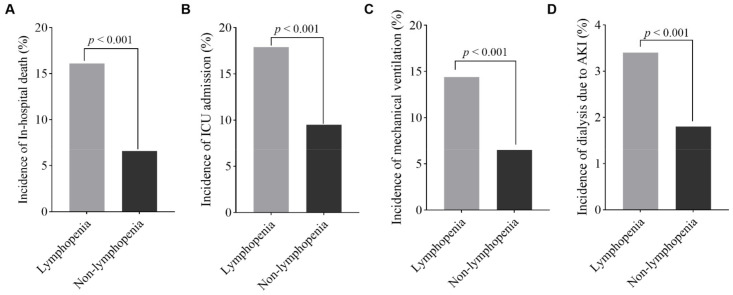
Comparison of the adverse outcomes in patients with or without lymphopenia upon hospital admission. Bar graphs show the incidences of in-hospital death (**A**), ICU admission (**B**), invasive mechanical ventilation (**C**), and dialysis due to AKI (**D**) in the study population classified into two groups according to their absolute lymphocyte counts at admission. Patients with lymphopenia had higher incidences of adverse outcomes (all *p* < 0.001).

Of the 4485 patients analyzed, 628 (14%) patients required admission to the ICU, 482 (10.7%) required invasive mechanical ventilation support, 119 (2.7%) required dialysis due to the development of AKI, and 538 (11.9%) hospitalized patients died during the study period. In comparison to patients who did not have lymphopenia on hospital admission, those with lymphopenia had higher incidences of ICU admission (17.9% versus 9.5%, *p* < 0.001), invasive mechanical ventilation (14.4% versus 6.5%, *p* < 0.001), dialysis (3.4% versus 1.8%, *p* < 0.001), and in-hospital mortality (16.6% versus 6.6%, *p* < 0.001), respectively (Figure 3). Patients with lymphopenia also had a longer hospital length of stay compared to those without lymphopenia (9 [IQR, 5–16] days versus 7 [IQR, 4–13] days; *p* < 0.001). 

### 3.4. Lymphopenia Is an Independent Risk Factor for Adverse Clinical Outcomes

**Figure 4:**
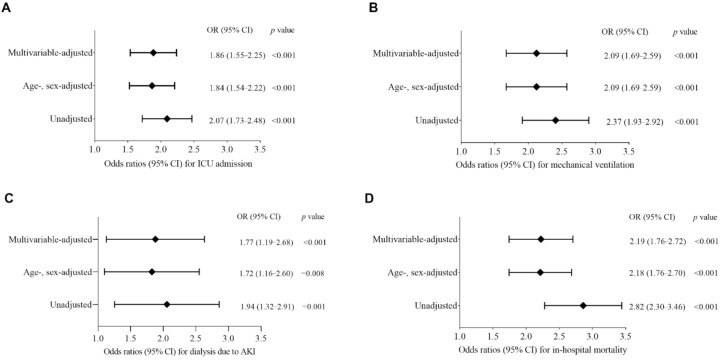
Forest plots showing association of lymphopenia with risks of adverse clinical outcomes in patients with COVID-19. Independent effects of lymphopenia (ALC < 1.1 × 10^9^/L) on the risks of ICU admission (**A**), invasive mechanical ventilation (**B**), dialysis due to AKI (**C**), and in-hospital mortality (**D**) during hospitalization were analyzed using logistic regression models and compared to individuals with ALC ≥ 1.1 × 10^9^/L. Results were reported as odds ratios (ORs) with 95% confidence intervals (CIs), adjusted for age-, sex-, and multi-variables including age, sex, race, diabetes, hypertension, obesity, smoking, COPD, CKD, CHD, and malignancy. ICU, intensive care unit; AKI, acute kidney injury.

Logistic regression models were used to measure the association of lymphopenia with adverse clinical outcomes in patients with COVID-19. The unadjusted model revealed a higher risk of adverse outcomes in patients with lymphopenia, of 2.07-fold for ICU admission (OR, 2.07; 95% CI, 1.73–2.48), 2.37-fold for invasive mechanical ventilation (OR, 2.37; 95% CI, 1.93–2.92), 1.94-fold for dialysis (OR, 1.94; 95% CI, 1.32–2.91), and 2.82-fold for in-hospital mortality (OR, 2.82; 95% CI, 2.30–3.46), compared with patients who did not present with lymphopenia (Figure 4). This pattern of association between lymphopenia and adverse outcomes persisted after adjusting for age and gender, with the adjusted ORs of 1.84 (95% CI, 1.54–2.22) for ICU admission, 2.09 (95% CI, 1.69–2.59) for invasive mechanical ventilation, 1.72 (95% CI, 1.16–2.60) for dialysis, and 2.18 (95% CI, 1.76–2.70) for in-hospital mortality, respectively (Figure 4). We then created a multivariable logistic regression model that included age, gender, race, and history of hypertension, diabetes, COPD, chronic kidney disease, coronary artery disease, malignancy, obesity, and smoking as covariates. After adjustment for these variables, the presence of lymphopenia was still found to be associated with the risks of adverse outcomes, with multivariable-adjusted ORs of 1.86 (95% CI, 1.55–2.25) for ICU admission, 2.09 (95% CI, 1.69–2.59) for invasive mechanical ventilation, 1.77 (95% CI, 1.19–2.68) for dialysis, and 2.19 (95% CI, 1.76–2.72) for in-hospital mortality, respectively, in comparison with individuals who did not have lymphopenia (Figure 4).

### 3.5. “Dose–Response” Relationships between Lymphopenia and Adverse Outcomes

**Figure 5:**
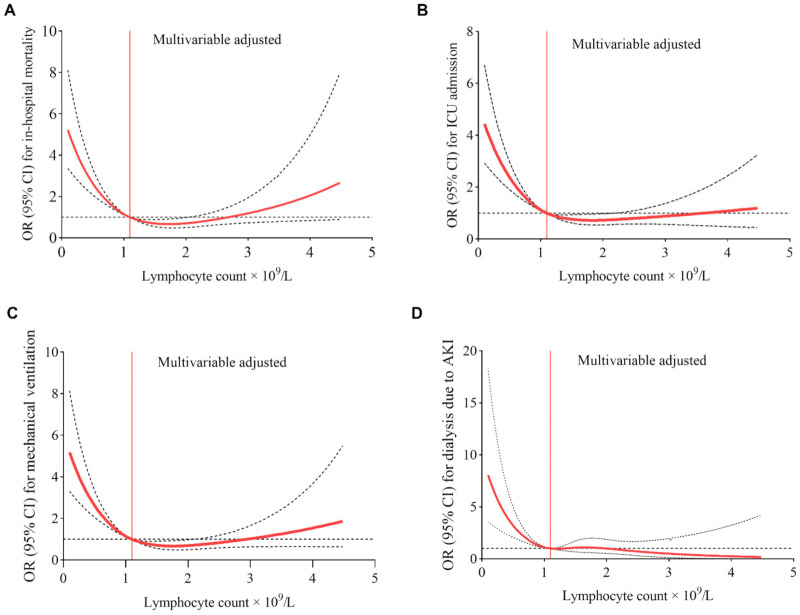
Dose–response association of absolute lymphocyte count and risk of the outcomes of interest in patients with COVID-19. Restricted cubic splines were generated using logistic regression models, and the lymphocyte value of 1.1 × 10^9^/L was set as reference (denoted by red vertical lines) for the continuous model adjusted to age, gender, race, and history of hypertension, diabetes, chronic obstructive pulmonary disease, chronic kidney disease, coronary artery disease, malignancy, obesity, and smoking. Solid red curves are ORs for the outcomes of interest (**A**–**D**), and dashed black lines indicate 95% confidence intervals based on fitting of cubic splines to risk estimates obtained using logistic regression.

The spline curves in Figure 5 illustrates the “dose–response” relationship between continuous changes in lymphocyte counts and changes in ORs for the risks of adverse outcomes, assessed using the RCS models adjusted for covariates including age, gender, race, and history of hypertension, diabetes, COPD, chronic kidney disease, coronary artery disease, malignancy, obesity, and smoking. When ALC levels were below 1.1 × 10^9^/L (lymphopenia), a negative nonlinear dose–response relationship was observed, with the highest ORs for adverse outcomes seen in patients with the lowest values of ALC. There was a sharp increase in the values of multivariable-adjusted ORs for in-hospital death, ICU admission, mechanical ventilation, and dialysis, with a decreasing of ALC, ultimately reaching corresponding ORs of 5.22, 4.44, 5.18, and 8.05, respectively at the lowest lymphocyte counts of 0.1 × 10^9^/L. When the ALC values were greater than or equal to 1.1 × 10^9^/L, multivariable-adjusted ORs for adverse outcomes did not differ from 1.0-reflecting the absence of a significant association between ALC and the risk of adverse outcomes.

### 3.6. Lymphopenia Predicts the Risk of Adverse Clinical Outcomes in COVID-19 Patients

**Figure 6:**
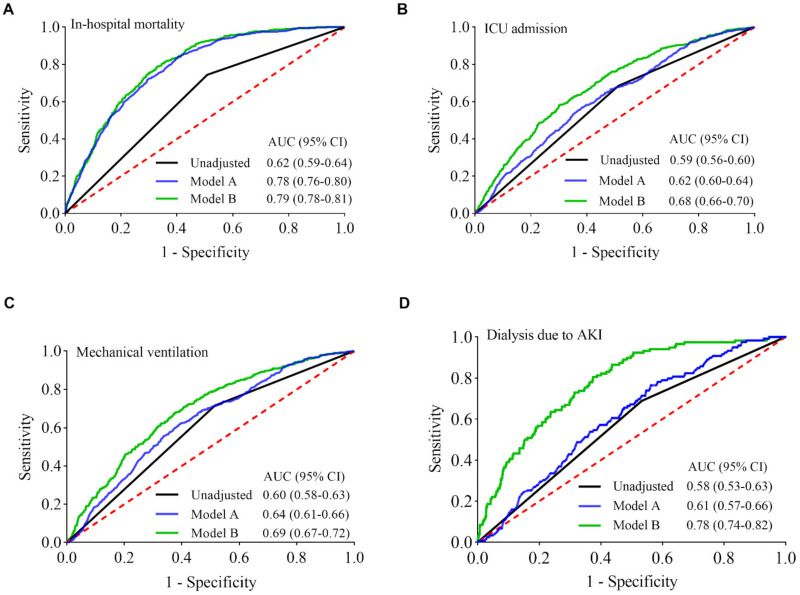
ROC curves of lymphopenia for predicting risks of the outcomes of interest. The areas under the ROC curves (AUCs) are values for estimating incidences of the outcomes of interest (**A**–**D**) through the use of logistic regression function calculated from unadjusted lymphopenia, model A, and model B, respectively. Model A, adjusted for age and gender; Model B, adjusted for age, gender, race, hypertension, diabetes, COPD, chronic kidney disease, coronary artery disease, malignancy, obesity, and smoking. Lymphopenia have the largest AUCs (areas under the green curves) to predict individual risk of adverse clinical outcomes with the incorporation of the known clinical risk factors. AKI, acute kidney injury; CI, confidence interval.

To explore lymphopenia as a tool to predict patients’ risk of adverse outcomes due to COVID-19 infection, receiver operating characteristic (ROC) curves and areas under ROC curves (AUCs) were calculated. Figure 6 (black curves) shows the ROC curves of lymphopenia for predicting risks of in-hospital mortality, ICU admission, invasive mechanical ventilation, and dialysis in COVID-19 patients, with the corresponding AUCs of 0.62 (95% CI, 0.59–0.64), 0.59 (95% CI, 0.56–0.60), 0.60 (95% CI, 0.58–0.63), and 0.58 (95% CI, 0.53–0.63), respectively. 

When taking age and gender into account, the predictive power of the combination of lymphopenia with age and gender in the risk model (model A) is superior to lymphopenia alone as a predictor of in-hospital mortality, as illustrated by the gap between two AUCs of 95% CIs (Figure 6A, blue and black curves; *p* < 0.001). There was no significant difference in the AUCs between lymphopenia alone and the combination of lymphopenia with age and gender in the risk models for ICU admission, invasive mechanical ventilation, and the need for dialysis due to AKI (Figure 6B–D, blue and black curves; *p* = 0.052, *p* = 0.103, and *p* = 0.327, respectively). Thus, the predictive power of the combination of lymphopenia with age and gender is similar to lymphopenia alone for predicting the risks of ICU admission, invasive mechanical ventilation, and dialysis. Utilizing an extensive combination of additional variables (age, gender, race, hypertension, diabetes, COPD, chronic kidney disease, coronary artery disease, malignancy, obesity, and smoking) together with the presence of lymphopenia in the predictive risk model (model B) enhances the predictive powers for the corresponding adverse outcomes, as demonstrated by the AUC values of 0.79 (95% CI, 0.78–0.81), 0.68 (95% CI, 0.66–0.70), 0.69 (95% CI, 0.67–0.72), 0.78 (95% CI, 0.74–0.82), respectively (Figure 6, green and black curves; all *p* < 0.001).

## 4. Discussion

COVID-19 is a highly infectious disease with significant morbidity and mortality. Gaining further insight into models predicting adverse clinical outcomes could help identify high-risk patients and prioritize workflow to optimize patient care. In this retrospective, population-based study of 4485 individuals, lymphopenia, defined as blood lymphocytes <1.1 × 10^9^/L, occurred in 53.7% of patients with COVID-19 at the time of admission. Patients with lymphopenia tended to be much older, male, and had higher rates of comorbid diseases than their non-lymphopenic counterparts. The lymphocyte counts were inversely associated with serum levels of CRP, IL-6, D-dimer, LDH, and creatinine. Lymphopenia was found to be an independent risk marker for ICU admission, invasive mechanical ventilation, dialysis, and in-hospital mortality. Moreover, a negative nonlinear dose–response relationship between lymphopenia and adverse outcomes was observed, with higher risks of adverse outcomes seen in patients with the lowest values of lymphocyte counts in patients with lymphopenia. Furthermore, the prognostic power of lymphopenia for adverse clinical outcomes were further strengthened by the incorporation of age, gender, race, and comorbidities. 

Lymphopenia has become a well-recognized risk marker for the severity of COVID-19 [21,22,23,26]. Lymphopenia is associated with a higher prevalence of the known risk factors of COVID-19 such as older age, male gender, hypertension, COPD, chronic kidney disease, coronary heart disease, and obesity [7,27,28,29]. This observation is well-documented in our data, showing a significantly higher comorbidity profile in the study population. Lymphopenia is also correlated with a hyper-inflammatory response [30], characterized by high serum levels of CRP, IL-6, D-dimer, and LDH, which is known to be related to the severity and worse outcomes of COVID-19 [8,9,31]. Data in our study supports these findings. We observed that the group of COVID 19 patients with lymphopenia had higher serum levels of pro-inflammatory mediators and higher rates of ICU admission, mechanical ventilation, dialysis due to AKI, and mortality, similarly seen in other COVID-19 studies [30,32,33]. Physiologically, a plausible mechanism linking lymphopenia to poor clinical outcomes specifically involves T lymphocytes. A higher total T cell count, including both CD4+ and CD8+, has been shown to be a predictor of a less severe disease and a more favorable clinical outcome in patients with COVID-19 [34]. In *The Lancet HIV*, two studies demonstrated worse outcomes in HIV patients with CD4 counts less than 200 cells per μL [35,36]. Overall, 70–80% of circulating lymphocytes are T lymphocytes and the underlying cause of lymphopenia in severe cases of COVID-19 has previously been attributed to apoptosis of T lymphocytes [37,38,39]. Mechanistically, SARS-CoV-2 infection can directly infect lymphocytes and induce cell death [40]. The generation of excessive pro-inflammatory cytokines, such as TNF-α and IL-6, can cause robust apoptosis of lymphocytes [41]. The apoptotic T lymphocytes can release the cognate ligand for Fas (FasL) that induces excessive apoptosis of epithelial cells and inflammation, resulting in pulmonary fibrosis [42,43]. Furthermore, SARS-CoV-2 not only diminishes the number of host’s lymphocytes but also induces an exhaustion of effector T cells and overproduction of pro-inflammatory cytokines, which in turn contributes to the disease progression and unfavorable outcomes. This observation is supported by the fact that administration of Tocilizumab, a specific anti-IL-6 monoclonal antibody, could increase the number of peripheral lymphocytes and reduce the pulmonary opacification in severe COVID-19 [44]. Therefore, preventing lymphopenia or restoring lymphocyte counts could represent a viable therapeutic opportunity in patients with severe COVID-19 [45,46,47]. Additional study is needed to characterize apoptosis in lymphocyte subsets and to explore the lines of cause and effect that may contribute to disease progression in COVID-19 patients.

Previous studies have explored lymphopenia in predicting clinical outcomes in patients with COVID-19 [23,26,33,48]. Although the definition of lymphopenia varied among studies, lymphopenia, defined as a lymphocyte count < 1.0 × 10^9^/L on admission, has been associated with the severity of disease and mortality from COVID-19 [33,48,49]. A subgroup analysis using a lymphocyte count < 1.5 × 10^9^/L to define lymphopenia showed a 3-fold increased risk of severe COVID-19 infection [26]. In this study, we set a cut-off point of <1.1 × 10^9^/L to define lymphopenia based on 4 prior studies using this cutoff point, previously demonstrating a consistent relationship between lymphopenia and the severity of COVID-19 [50,51,52]. We found that lymphopenia is relatively common with 53.7% of patients having lymphopenia on presentation-comparable prevalence to that of large-sized cohort studies from the United States [53,54] and China [55]. Lymphopenia was consistently found to be independently associated with adverse clinical outcomes, namely ICU admission, the use of mechanical ventilation, dialysis and in-hospital mortality. These data are consistent with prior findings showing an increased risk for worse outcomes among patients with lymphopenia [21,33,51,55,56,57,58]. Our study provides additional data about a negative nonlinear dose–response relationship between lymphopenia and risks for the adverse outcomes, highlighting a higher risk profile for adverse outcomes in patients with the lowest values of ALC at the time of admission [21]. The ROC analyses reveal that utilizing an extensive combination of additional variables together with the presence of lymphopenia in the predictive risk model enhances the predictive ability of the model for adverse outcomes.

The present study is limited by being a single center, retrospective cohort study. Selection bias and other limitations may exist—and all potential confounders may have not been included in the multivariable adjusted analyses. In this study, we aimed to focus on admission findings as predictive markers for adverse outcomes, hence our multivariable adjusted analyses did not include the type and timing of treatments as variables, which may impact the clinical outcomes in COVID-19 patients. For instance, corticosteroid use in patients with lymphocyte counts ≤ 0.82 × 10^9^/L was associated with a significantly reduced risk of all-cause mortality, whereas no significant difference was found in patients with lymphocytes > 0.82 × 10^9^/L receiving corticosteroids [59]. A meta-analysis of 44 studies comprising 20,197 patients demonstrates a beneficial effect of corticosteroids on short-term mortality and a reduction in the need for mechanical ventilation [60]. In addition, ALC was assessed on admission to hospital only. We did not evaluate the impact of dynamic changes of lymphocyte counts on clinical outcomes. Additionally, we did not measure specific lymphocyte subsets; thus, we do not know the degree to which lymphopenia was pronounced in each subset of lymphocytes, which represents important data that may provide further insight into disease severity and outcomes of COVID-19 hospitalized patients. Despite these limitations, our findings were internally consistent and in agreement with the previous published studies [21,22,26,33,49].

## 5. Conclusions

Lymphopenia, defined as ALC < 1.1 × 10^9^/L, is an independent risk factor associated with adverse outcomes in hospitalized COVID-19 patients. Patients with lymphopenia with the lowest ALC values are more likely to have a poor prognosis. Early recognition of this immunological phenotype could assist in the timely recognition and prognostication of severe COVID-19 patients. As lymphocytes are a pivotal component of the host immune responses to SARS-CoV-2 infection, our results highlight that targeting lymphocytes could be a promising strategy in the treatment of SARS-CoV-2 virus infection.

## Figures and Tables

**Table 1:** Baseline characteristics of patients stratified by absolute lymphocyte count.

Variables	Total Patients	Absolute Lymphocyte Count		*p* Value
		<1.1 × 10^9^/L	≥1.1 × 10^9^/L	
Patients, n (%)	4485	2409 (53.7)	2076 (46.3)	
Age, years	60 (46–72)	64 (52–75)	57 (41–70)	<0.001
Male, n (%)	2311 (51.5)	1413 (58.7)	898 (43.3)	<0.001
Race, n (%)				<0.001
White	756 (16.9)	451 (18.7)	305 (14.8)	
Black	1358 (30.3)	593 (24.6)	765 (36.8)	
Hispanic	2146 (47.8)	1244 (51.6)	902 (43.4)	
Asian	49 (1.1)	31 (1.3)	18 (0.9)	
Other	176 (3.9)	90 (3.7)	86 (4.1)	
Comorbidity, n (%)				
Diabetes	1813 (40.4)	1000 (41.5)	813 (39.2)	0.109
Hypertension	2953 (65.8)	1701 (70.6)	1252 (60.3)	<0.001
COPD	374 (8.3)	234 (9.7)	140 (6.7)	<0.001
Chronic kidney disease	670 (14.9)	399 (16.6)	271 (13.1)	<0.001
Coronary heart disease	705 (15.7)	420 (17.4)	285 (13.7)	<0.001
Malignancy	231 (5.2)	136 (5.7)	95 (4.6)	0.106
Obesity	1061 (23.7)	835 (34.7)	226 (10.9)	<0.001
Smoking	1089 (24.3)	623 (25.9)	466 (22.4)	0.008
Laboratory testing				
CRP, mg/L	6.6 (2.8–12.1)	7.4 (3.4–12.9)	5.6 (2.2–10.9)	<0.001
D-dimer, mg/L	0.9 (0.5–1.8)	0.9 (0.5–1.9)	0.9 (0.5–1.8)	0.142
IL-6, mg/L	36.2 (13.4–85.9)	39.8 (14.7–94.2)	30.6 (12.2–73.8)	0.019
LDH, U/L	317 (243–432)	337 (258–450)	293 (226–398)	<0.001
Creatinine, mg/L	0.9 (0.7–1.2)	0.9 (0.7–1.2)	0.8 (0.6–1.1)	<0.001

Data given as median and interquartile range (IQR) or *n* (%); COPD, chronic obstructive pulmonary disease; CRP, C-reactive protein; IL-6, interleukin-6; LDH, lactate dehydrogenase.

## Data Availability

Data can be obtained from the corresponding author upon reasonable request.
